# Change in Brainstem Gray Matter Concentration Following a Mindfulness-Based Intervention is Correlated with Improvement in Psychological Well-Being

**DOI:** 10.3389/fnhum.2014.00033

**Published:** 2014-02-18

**Authors:** Omar Singleton, Britta K. Hölzel, Mark Vangel, Narayan Brach, James Carmody, Sara W. Lazar

**Affiliations:** ^1^Massachusetts General Hospital, Harvard Medical School, Boston, MA, USA; ^2^Institute for Medical Psychology, Charité Universitätsmedizin, Berlin, Germany; ^3^PGSP-Stanford Psy.D. Consortium, Palo Alto University, Palo Alto, CA, USA; ^4^University of Massachusetts Medical School, Worcester, MA, USA

**Keywords:** brain stem, mindfulness, well-being, stress, psychological, raphe nuclei

## Abstract

Individuals can improve their levels of psychological well-being (PWB) through utilization of psychological interventions, including the practice of mindfulness meditation, which is defined as the non-judgmental awareness of experiences in the present moment. We recently reported that an 8-week-mindfulness-based stress reduction (MBSR) course lead to increases in gray matter concentration in several brain areas, as detected with voxel-based morphometry of magnetization prepared rapid acquisition gradient echo MRI scans, including the pons/raphe/locus coeruleus area of the brainstem. Given the role of the pons and raphe in mood and arousal, we hypothesized that changes in this region might underlie changes in well-being. A subset of 14 healthy individuals from a previously published data set completed anatomical MRI and filled out the PWB scale before and after MBSR participation. PWB change was used as the predictive regressor for changes in gray matter density within those brain regions that had previously shown pre- to post-MBSR changes. Results showed that scores on five PWB subscales as well as the PWB total score increased significantly over the MBSR course. The change was positively correlated with gray matter concentration increases in two symmetrically bilateral clusters in the brainstem. Those clusters appeared to contain the area of the pontine tegmentum, locus coeruleus, nucleus raphe pontis, and the sensory trigeminal nucleus. No clusters were negatively correlated with the change in PWB. This preliminary study suggests a neural correlate of enhanced PWB. The identified brain areas include the sites of synthesis and release of the neurotransmitters, norepinephrine and serotonin, which are involved in the modulation of arousal and mood, and have been related to a variety of affective functions as well as associated clinical dysfunctions.

## Introduction

Mindfulness meditation, a practice with origins in ancient Buddhist meditation traditions, has long been reported to produce positive effects on psychological well-being (PWB) that extend beyond the time the individual is actually meditating (Ekman and Davidson, 1994; Baer, 2003; Ekman et al., 2005). Taking advantage of these benefits, mindfulness practices have been increasingly incorporated into psychotherapeutic programs over the last three decades (Kabat-Zinn, 1990; Linehan, 1993; Roemer and Orsillo, 2002; Segal et al., 2002; Luoma et al., 2008). Mindfulness is defined as the purposeful and non-judgmental awareness of present-moment experience (Kabat-Zinn, 2003).

Research has shown that interventions incorporating mindfulness training positively affect symptoms of a variety of disorders including anxiety (Roemer et al., 2009; Hofmann et al., 2010), depression (Teasdale et al., 2000; Hofmann et al., 2010), and attention deficit hyperactivity disorder (Zylowska et al., 2008; van de Weijer-Bergsma et al., 2012). Furthermore, preliminary evidence suggests that mindfulness-based interventions can positively influence sleep and dietary patterns in clinical populations (Baer et al., 2005; Winbush et al., 2007; Dalen et al., 2010).

Recently, neuroimaging studies have begun to explore changes in neural structure and function associated with meditation practice (Davidson, 2003; Lazar et al., 2005; Brefczynski-Lewis et al., 2007; Farb et al., 2007; Pagnoni and Cekic, 2007; Slagter et al., 2007; Hölzel et al., 2008; Lutz et al., 2008). A number of anatomical MRI studies have demonstrated that individuals who have regularly practiced meditation for several years exhibit a different gray matter morphometry in multiple brain regions when compared to demographically matched controls (Lazar et al., 2005; Pagnoni and Cekic, 2007; Hölzel et al., 2008; Luders et al., 2009; Vestergaard-Poulsen et al., 2009). Recently, we reported the first longitudinal study of gray matter changes following an 8-week-mindfulness-based stress reduction (MBSR) course (Hölzel et al., 2011). One region with enhanced gray matter concentration following the MBSR course was in the cerebellar vermis, reaching into a region of the brain stem that included the locus coeruleus, nucleus raphe pontis, pontine tegmentum, and the sensory trigeminal nucleus (Naidich et al., 2008). The locus coeruleus, a site of synthesis of norepinephrine, has been implicated in conditions such as depression and anxiety (Aston-Jones and Cohen, 2005). Furthermore, this region may play a role in modulating serotonin release (Plaznik and Kostowski, 1983; Grenhoff et al., 1993; Ressler and Nemeroff, 1999). The modulation of levels of serotonin, which is synthesized in the raphe nuclei, has been shown to be one of the most effective treatments for mood and anxiety disorders (Masand and Gupta, 2002). The pontine tegmentum, part of the cholinergic system, is implicated in regulating selective attention, wakefulness, learning, reward, and sleep (Kobayashi and Okada, 2007; Wang and Morales, 2009).

Given that these regions are well-known to modulate several systems, including the serotonin, dopamine, and norepinephrine systems, as well as play central roles in processes such as mood, arousal, sleep, and appetite (Ressler and Nemeroff, 1999; Aston-Jones and Cohen, 2005; Winn, 2006; Kobayashi and Okada, 2007; Wang and Morales, 2009; Bailer and Kaye, 2011), we reasoned that gray matter changes in these regions might contribute to enhanced well-being following mindfulness practice. A subset of individuals in our previous study had completed a questionnaire to assess PWB. Therefore, in order to test this hypothesis, we re-analyzed this subgroup of the previous data set and investigated correlations between changes in gray matter concentration and changes in self-report measures of PWB.

## Materials and Methods

### Participants

The PWB scale was administered to a subsample of 14 participants from our previous study (Hölzel et al., 2011). As described previously (Hölzel et al., 2011), participants were recruited from MBSR courses held at the Center for Mindfulness at the University of Massachusetts Medical School. Individuals were included if they presented as physically and psychologically healthy, scored ≥1 SD above the population mean on the four-item Perceived Stress Scale (PSS; Cohen and Williamson, 1988), had no significant previous meditation experience, were between 25 and 50 years old, had no contra-indications for MRI scanning (i.e., metallic implants, claustrophobia, pregnancy), and made a verbal commitment to attend all eight classes and perform the prescribed daily meditation exercises.

The participants were healthy, right-handed individuals [five male and nine female; mean age: 37.9 years (SD: 4.3 years; age range: 29–44 years)]. Participants had an average of 17.5 years of education (SD: 1.9 years). Ethnicities were: 11 Caucasians, 1 South Asian, 1 African American, 1 multi-ethnic. Participants received a $300 discount in the MBSR course fee (which costs between $475 and $630, depending on the household income) for their participation in the study. Additional analyses that included data from this sample have been reported elsewhere (Hölzel et al., 2010, 2011). The study protocol was approved by the IRBs of Massachusetts General Hospital and the University of Massachusetts Medical School and written informed consent was obtained from all participants.

### MRI data collection and analysis

Participants were scanned at the Martinos Center for Biomedical Imaging in Charlestown, MA, USA, during the 2 weeks before (Pre) and after (Post) participation in MBSR. High-resolution MRI data were acquired with a Siemens Magnetom Avanto 1.5 T scanner with standard head coil. Data sets of the whole brain were collected using a T1 weighted, magnetization prepared rapid acquisition gradient echo (MPRAGE) sequence, consisting of 128 sagittal slices (voxel size: 1.0 mm × 1.0 mm × 1.3 mm, TI = 1000 ms; TE = 3.39 ms; TR = 2730 ms, flip angle 7°, matrix 256 mm × 256 mm). Image analysis was performed with VBM tools within the SPM5 neuroimaging statistical software (Wellcome Department of Cognitive Neurology, London, www.fil.ion.ucl.ac.uk/spm/software/spm5/) based in MATLAB 7.1, release 14 (Mathworks Inc., Natick, MA, USA), using default settings unless otherwise specified. Images were manually aligned to the anterior commissure and then segmented into gray and white matter in native space (i.e., before normalization, using the “Native Space” segmentation option implemented in SPM5). For each individual, the (unmodulated) gray matter segmentations of the Pre and Post images were spatially coregistered. Normalization parameters were calculated for the Pre scan and were applied to both time-points (trilinear interpolation, 2 mm × 2 mm × 2 mm), to make sure that regional differences between the images were not removed by scan-specific spatial normalization (Driemeyer et al., 2008; Ilg et al., 2008). Images were smoothed using an 8-mm full width at half maximum Isotropic Gaussian Kernel.

### Psychological well-being

Psychological well-being was assessed using the 54-item version of the PWB scale (PWB) by Ryff (1989). The PWB is based on a model comprising six factors of PWB (Ryff, 1989; Ryff and Keyes, 1995): self-acceptance (positive attitude toward oneself even while aware of one’s own limitations), positive relations with others (developing and maintaining warm and trusting interpersonal relationships), environmental mastery (managing one’s environment so as to meet personal needs and desires), autonomy (sense of self-determination and personal authority), purpose in life (sense of meaning in one’s effort and challenges), and personal growth (view of self as growing and developing, openness to new experiences). These six factors integrate into a single second-order factor (Ryff and Keyes, 1995). The 54-item version of the PWB scale has been shown to have good psychometric properties (Sewell et al., 2004). Items were rated on a six-point continuum ranging from strongly disagree to strongly agree. The total score is derived by summing the scores on the six factors.

In a regression analysis using SPM5, Pre- to Post-intervention changes in the PWB total score were correlated with changes in gray matter concentration in regions that have previously been identified as showing an increase in gray matter concentration over the 8-week-MBSR course (Hölzel et al., 2011). In the previous study, we had identified these regions by performing a paired *t*-test within the group that had undergone the MBSR program, choosing a cluster-size threshold that was corrected for multiple comparisons across the entire brain (i.e., in order to exceed the threshold of *p* < 0.05, clusters had to exceed a size of 250 voxels) and based on statistical parametric maps with an initially, uncorrected, thresholded of *p* = 0.01. For the current study, we created a mask that contained the result of that previous study as our new region of interest. To be conservative, we included into this mask all the clusters that displayed a significant increase in gray matter concentration from Pre- to Post-intervention (cf. Table 2 and Figure 2 in Hölzel et al., 2011), namely the clusters within the brainstem/cerebellum, PCC, and left TPJ, i.e., four clusters with a total of 1537 voxels. To obtain images representing the change in gray matter concentration, the Pre-intervention scan was subtracted from the Post-intervention scan. Cluster level statistics for the current analysis are reported on an alpha level of <0.05, multiple comparisons corrected for the search region (height threshold: *p* = 0.01).

## Results

### Improvements in psychological well-being

A paired-samples *t*-test revealed a significant increase in PWB from Pre- to Post-intervention (Pre mean: 224.64, SD: 28.62; Post mean: 252.75, SD: 26.89; *t* = −4.03; *p* = 0.001). Pre-Post changes for five of the six scales were also significant: self-acceptance (mean pre: 34.18, SD: 8.18, mean post: 40.93, SD: 6.67, *t* = −4.21, *p* = 0.001), environmental mastery (mean pre: 31.68, SD: 5.78, mean post: 36.68, SD: 6.47, *t* = −2.90, *p* = 0.012), autonomy (mean pre: 40.07, SD: 7.41, mean post: 44.14, SD: 6.15, *t* = −2.97, *p* = 0.011), purpose in life (mean pre: 38.14, SD: 8.02, mean post: 43.14, SD: 4.93, *t* = −2.66, *p* = 0.020), and personal growth (mean pre: 43.21, SD: 4.98, mean post: 47.21, SD: 4.93, *t* = −3.61, *p* = 0.003). The sixth scale, positive relations with others, revealed a trend toward significance (mean pre: 37.36, SD: 8.85, mean post: 40.64, SD: 7.04, *t* = −1.87, *p* = 0.084). When applying the very conservative Bonferroni multiple comparison correction for the six sub-tests, pre-post changes for the scales self-acceptance and personal growth remained significant, but all other subscales missed significance.

### Correlation between changes in psychological well-being and changes in gray matter concentration

**Figure 1:**
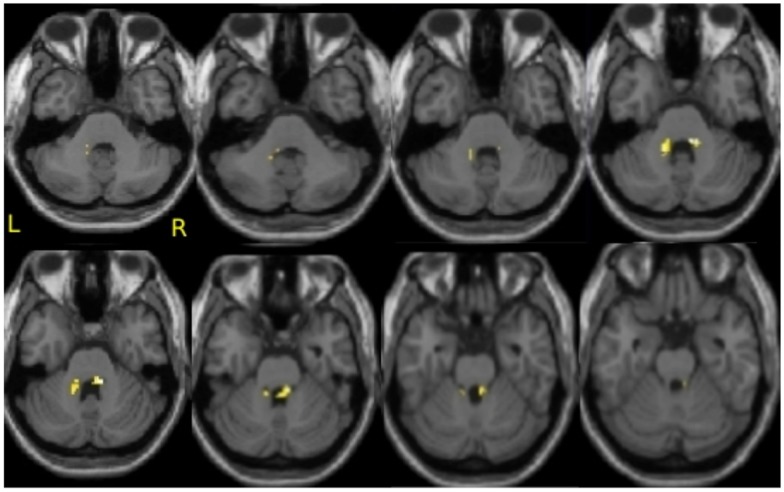
**Correlation of improvements in psychological well-being and increase in gray matter concentration in the brainstem**. Axial slices from *z* = −38 to −24, with an image every 2 voxels. Significant clusters are overlaid over the group averaged normalized structural MPRAGE image. This analysis includes *N* = 14 participants.

**Figure 2:**
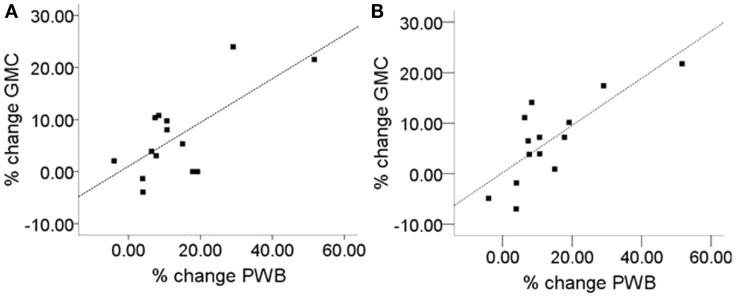
**Correlation of improvement of psychological well-being (PWB) and change in gray matter concentration (GMC)**. **(A)** Left brainstem cluster (peak *x*, *y*, *z*: −14, −42, −32; cluster-size *k*: 37), Pearson correlation coefficient: *r* = 0.72), **(B)** right brainstem cluster (peak *x*, *y*, *z*: 12, −36, −30; cluster size *k*: 43; Pearson correlation coefficient: *r* = 0.76). For the two regions displayed in Figure 1, values were averaged across the cluster and plotted against the change in PWB. Units indicate the percent change relative to the Pre-intervention baseline.

To address the question of whether increase in gray matter concentration were related to improvements in well-being, the change in the total PWB score was regressed against changes in gray matter concentration within the regions identified in Hölzel et al. (2011). Within the chosen mask, two clusters in the brainstem were identified to be positively correlated with changes in PWB [Figure 1; right cluster: cluster-size *k*: 43 voxels; *p* = 0.024; MNI coordinates of peak voxel (*x*, *y*, *z*): 12, −36, −30; left cluster: cluster-size *k*: 37 voxels; *p* = 0.040; MNI coordinates of peak voxel (*x*, *y*, *z*): −14, −42, −32]. The more the participants’ PWB improved over the 8-week-MBSR course, the more increase in gray matter concentration was observed in these regions. According to the atlas by Naidich et al. (2008), these clusters appear to contain the area of the pontine tegmentum, locus coeruleus, nucleus raphe pontis, and the sensory trigeminal nucleus bilaterally. For illustrative purposes, values were extracted and averaged across each cluster and plotted with the change in PWB total score (Figure 2). The Pearson coefficients were 0.72 (*p* = 0.004) for the correlation between PWB change and change in the left brainstem cluster, and 0.76 (*p* = 0.002) for the correlation between PWB change and change in the right brainstem cluster. Importantly, these numbers are reported only for comparative purposes, and should not be interpreted by themselves, since clusters were derived through searching for correlations with PWB scores in the first place. Using the Tukey-criterion of defining outliers as those values that are further than 1.5 times the interquartile range away from the upper or lower quartile (Tukey, 1977), we identified one single outlier, namely the individual with the highest change in PWB total score. When excluding this outlier from the analysis, the correlation coefficients dropped slightly, but remained significant for the right cluster (*r* = 0.716, *p* = 0.006) and almost significant for the left cluster (*r* = 0.553, *p* = 0.050). When additionally excluding the individual with the second highest change in PWB total score, the correlation with the left brainstem cluster was no longer significant (*r* = 0.165, *p* = 0.609), but the correlation with the cluster in the right side of the brainstem remained significant (*r* = 0.59, *p* = 0.043). No clusters or voxels were negatively correlated with the change in PWB. No clusters were negatively correlated with the change in PWB.

## Discussion

We identified a positive correlation between improvement in PWB and increase in gray matter concentration within regions of the brainstem, suggesting that these morphological changes might be part of a mechanism underlying the changes in PWB. Regions within the brainstem were found to increase in gray matter concentration over the 8 weeks (Hölzel et al., 2011), and the increase within a sub-region of the original area of change was correlated with improvements in PWB. These regions appear to include the area of the locus coeruleus, pontine tegmentum, nucleus raphe pontis, and the sensory trigeminal nucleus (DeArmond et al., 1989; Naidich et al., 2008). Several previous cross-sectional studies have investigated the impact of meditation practice on brain morphology by comparing groups of experienced meditators to non-meditators (Lazar et al., 2005; Pagnoni and Cekic, 2007; Hölzel et al., 2008; Luders et al., 2009; Vestergaard-Poulsen et al., 2009). None of these previous studies assessed the participants’ PWB, and all used a cross-sectional study design, which is usually not as sensitive as a longitudinal design and which suffers from well-known limitations (i.e., the possibility of pre-existing conditions or other life-style differences which may confound results).

The raphe nuclei are a major site of serotonergic neurons (Michelsen et al., 2007), which project widely throughout the brain. Serotonin is implicated in numerous functions including sleep (Monti and Monti, 2000; Monti, 2011), mood, appetite, and conditioned fear. Further, stress has been shown to downregulate serotonin receptors in the raphe nuclei (Fuchs and Flügge, 2003). Modulation of the serotonin system has been profoundly effective for the treatment of a wide range of mood and anxiety disorders (Masand and Gupta, 2002) and the serotonergic neurons of the dorsal raphe nuclei have been implicated in eating disorders (Bailer et al., 2007; Bailer and Kaye, 2011). Interestingly, mindfulness training has been shown to improve a number of conditions for which altered serotonin levels have been implicated, including anxiety and depression (Teasdale et al., 2000; Baer, 2003; Kuyken et al., 2008; Roemer et al., 2009), insomnia (Kuyken et al., 2008; Ong et al., 2009), eating disorders (Kristeller et al., 2006), as well as improvements in sleep patterns (Carlson et al., 2003; Carlson and Garland, 2005; Ong et al., 2009), and attention (Jha et al., 2007). Considering the importance of serotonin for a number of factors that contribute to PWB including mood and sleep patterns, our findings of increased gray matter concentration in the raphe following MBSR that is correlated with improved well-being is highly suggestive.

The locus coeruleus is the site of synthesis and release of the neurotransmitter norepinephrine, and is thought to optimize behavioral performance by modulating arousal, regulating the interplay between focused vs. flexible responding to environmental demands, or selective vs. scanning attention (Aston-Jones et al., 2000, 2007; Aston-Jones and Cohen, 2005). The neurons of the locus coeruleus are important in a variety of cognitive, affective, and other behavioral functions, as well as associated clinical dysfunctions (e.g., depression, anxiety, sleep, and circadian disorders; for discussion, see Aston-Jones et al., 2007). It is also one of the primary sites mediating the stress response as well as a site of action of antidepressant drugs (Brady, 1994). This may be related to the influence of the norepinephrine system on perceptions of personal control and autonomy (Bandura et al., 1985; Ryff et al., 2006), which our results (autonomy and environmental mastery subscales) show are improved following MBSR participation. Compared to healthy controls, depressed individuals display reduced gray matter density in this region (Chan-Palay and Asan, 1989; Arango et al., 1996; Ressler and Nemeroff, 1999). Norepinephrine is thought to act as a modulatory agent, modulating serotonin and dopamine release through projections into the ventral tegmental area and the dorsal raphe nuclei (Plaznik and Kostowski, 1983; Grenhoff et al., 1993; Ressler and Nemeroff, 1999). Furthermore, there is evidence that bias toward negative memories and emotions in depression may be related to norepinephrine, and that potentiation of norepinephrine results in increased recognition of positive emotions and more positive emotional bias (Harmer et al., 2003). Several studies have found changes in serum concentration of serotonin and norepinephrine, particularly decrease in norepinephrine and increase in serotonin in meditators (Infante et al., 2001; Solberg et al., 2004; Curiati et al., 2005; Yu et al., 2011).

The pontine tegmentum and its nuclei, the pedunculopontine nucleus and the laterodorsal tegmental nucleus, are also part of the brain’s cholinergic system, and have been indicated as working as a modulatory system influencing learning, reward, sleep/wakefulness, motor function, and attention (Kobayashi and Okada, 2007; Wang and Morales, 2009). The pedunculopontine nucleus and laterodorsal tegmental nucleus neurons send axons to dopamine-containing areas of the ventral tegmental area and the substantia nigra, as well as to the lateral hypothalamus, thalamus, and basal ganglia, wherein glutamine and acetylcholine may act to modulate reward and learning (Yeomans et al., 1993; Steiniger and Kretschmer, 2003). In addition, the pedunculopontine nucleus may play a role in associative learning and reward as a relay for contextual information to midbrain dopamine neurons (Pan and Hyland, 2005). Executive control processes contribute to PWB (e.g., autonomy, environmental mastery; Ryff et al., 2006) and the pontine tegmentum has also been implicated in REM sleep (Fuller et al., 2007), which also correlates positively with well-being (Ryff et al., 2006).

This study comes with several important limitations: first, the sample size is extremely small, and findings are therefore unreliable. Second, with a relatively low resolution of the acquired images and additional smoothing, spatial sensitivity is limited, which is especially relevant when looking at small nuclei. Third, it has been discussed in the literature that segmentation and normalization of the brainstem is particularly problematic (Beissner et al., 2011). The exact localization of the regions identified here therefore needs to be confirmed. Fourth, the brainstem search territory was defined by our previous between-group analysis, and as such might be considered “non-independent” from the current correlation analysis (e.g., Vul et al., 2009, but see also Lieberman et al., 2009; Poldrack and Mumford, 2009). As a consequence, we would like the results of this study to be understood as purely speculative, and hope that they might be used to generate hypotheses for future rigorous research.

An extensive body of research during the last decade has established that MBSR leads to improvements in psychological health and well-being (Grossman et al., 2004; Nyklícek and Kuijpers, 2008; Shapiro et al., 2008). Interestingly, the data presented here suggest well-being is associated with brainstem regions that are the primary production sites of several neurotransmitters and which modulate basic functions of survival (sleep, appetite) and mood/arousal. Given that chronic stress increases the likelihood of developing future psychopathology, knowledge of the neurobiological mechanisms of behavioral interventions used in reducing stress and promoting well-being will be of great clinical interest.

## Conflict of Interest Statement

The authors declare that the research was conducted in the absence of any commercial or financial relationships that could be construed as a potential conflict of interest.
